# Phytochemicals and Biological Activities of *Garcinia morella* (Gaertn.) Desr.: A Review

**DOI:** 10.3390/molecules25235690

**Published:** 2020-12-02

**Authors:** Hosakatte Niranjana Murthy, Dayanand Dalawai, Yaser Hassan Dewir, Abdullah Ibrahim

**Affiliations:** 1 Department of Botany, Karnatak University, Dharwad 580003, India; hnmurthy60@gmail.com (H.N.M.); dayananddalawai@gmail.com (D.D.); 2 Plant Production Department, College of Food and Agriculture Sciences, King Saud University, P.O. Box 2460, Riyadh 11451, Saudi Arabia; adrahim@ksu.edu.sa; 3 Faculty of Agriculture, Kafrelsheikh University, Kafr El-Sheikh 33516, Egypt

**Keywords:** bioactive compounds, benzophenone, flavonoids, xanthones

## Abstract

*Garcinia morella* (Gaertn.) Desr. is an evergreen tree that yields edible fruits, oil, and resin. It is a source of “gamboge”, a gum/resin that has a wide range of uses. The fruits, leaves, and seeds of this tree are rich in bioactive compounds, including xanthones, flavonoids, phenolic acids, organic acids, and terpenoids. Evidence from different studies has demonstrated the antioxidant, antifungal, antiviral, hepatoprotective, anticancer, anti-inflammatory, antibacterial, and larvicidal activities of the fruit, leaf, and seed extracts of *G. morella*. This review summarizes the information on the phytochemicals of *G. morella* and the biological activities of its active constituents.

## 1. Introduction

**Figure 1:**
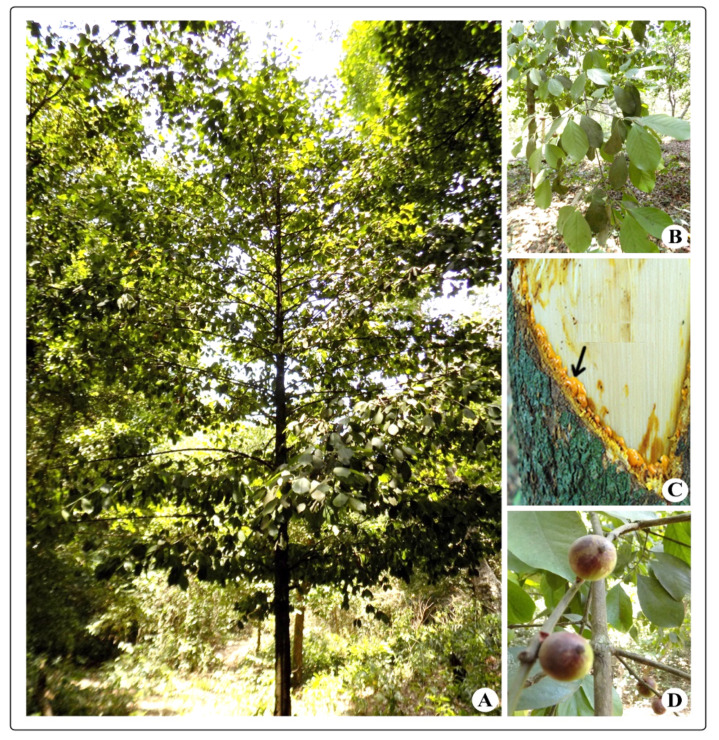
*Garcinia morella*: (**A**) tree; (**B**) leaves; (**C**) resin from bark; (**D**) fruits.

*Garcinia morella* (Gaertn.) Desr., known as Indian gamboge, is a fruit-yielding tree belonging to the family Clusiaceae and is a close relative of mangosteen (*G. mangostana*). It is an evergreen tropical tree naturally distributed across the Indian subcontinent to Indochina and Sri Lanka. In India, it is commonly distributed in the Western Ghats and northeastern regions. The tree grows up to 12 m tall (Figure 1A); leaves are simple, opposite, and decussate (Figure 1B); and bark is smooth and dark brown with white blaze, which oozes out a gum/resin that is bright yellow in color (Figure 1C). Fruits are berries with a diameter of 3 cm that contain four seeds (Figure 1D). Fruits are esteemed as a dessert fruit and are preserved by slicing and sun-drying. The yellow fat obtained from the seed is used in cooking and confectionery [1]. It is also used as a substitute for ghee. Gamboge, the gum/resin obtained from the plant, is used as a yellow dye, as an illuminant, and in varnishes and watercolors. It is traditionally collected by cutting a thin slice off the bark of the tree about the size of the palm of the hand; the resin collects there and is scraped off when sufficiently dried. The plant is sometimes used as a root stock for mangosteen (*G. mangostana*) [2].

## 2. Phytochemicals Isolated from *G. morella*

Indian gamboge has been reported to contain diverse secondary metabolites that have been primarily isolated from the leaves, fruits, seeds, resin, and heartwood of the plant (Table 1). The major isolated phytochemicals were xanthones, benzophenone/s, flavonoids, phenolic acids, organic acids, triterpenoids, and fatty acids (Table 1, [3,4,5,6,7,8,9,10,11,12,13,14]).

### 2.1. Xanthones

**Figure 2:**
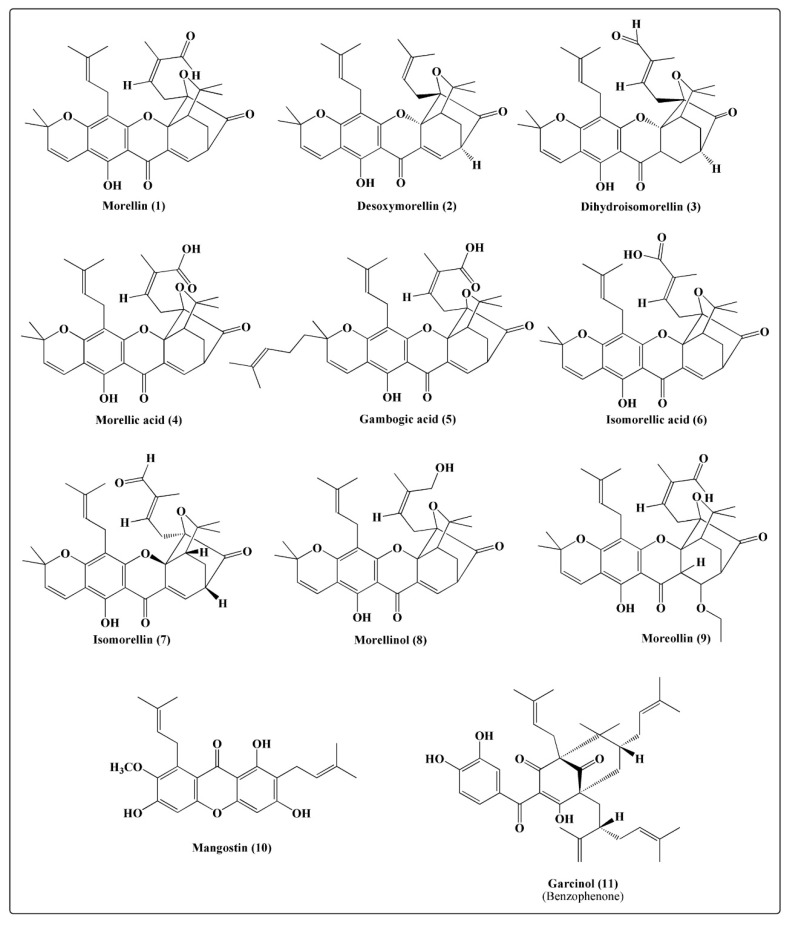
Structures of some xanthones and benzophenone compounds isolated from *Garcinia morella.*

Xanthones are heterocyclic compounds having a dibenzo-γ-pyrone skeleton as the basic structure. They constitute the primary compounds of lichens and have also been reported in microorganisms [15]. Xanthones are classified into the following five major groups: simple oxygenated xanthones, xanthone glycosides, prenylated and related xanthones, xanthonelignoids, and miscellaneous xanthones [16]. Xanthones have been abundantly reported in the angiosperm families, namely Gentianaceae, Clusiaceae, Moraceae, and Polygonaceae. More than 70 xanthones have been reported from *Garcinia* species, especially from the mangosteen fruit [17]. The first xanthone isolated from Indian gamboge seeds was morellin (**1**) [6,7]. Desoxymorellin (**2**), dihydromorellin (**3**), and isomorellin (**7**) were isolated from its seeds and resin previously [5,12]. Gambogic acid (**5**) was isolated from leaves and resin [3,10,11]. Later, morellinol (**8**) and moreollin (**9**) were isolated from the seeds of Indian gamboge [8,9]. Similarly, isomorellic acid (**6**) and morellic acid (**4**) were extracted from its resin [11]. Recently, mangostin (**10**) was isolated from the leaves of Indian gamboge [3]. These compounds are detailed in Figure 2.

### 2.2. Benzophenones

Benzophenones are a class of compounds that have a common phenol-carbonyl-phenol skeleton and exhibit significant structural diversity. Plant species belonging to the family Clusiaceae are characterized by the presence of benzophenones [18]. Various polyisoprenylated benzopheones have been reported from *G. mangostana*, *G. indica*, and *G. gummigutta* [19]. Garcinol (**11**) (Figure 2, Table 1) was isolated and identified from the fruits and leaves of Indian gamboge [3,13].

### 2.3. Flavonoids and Phenolic Acids

**Figure 3:**
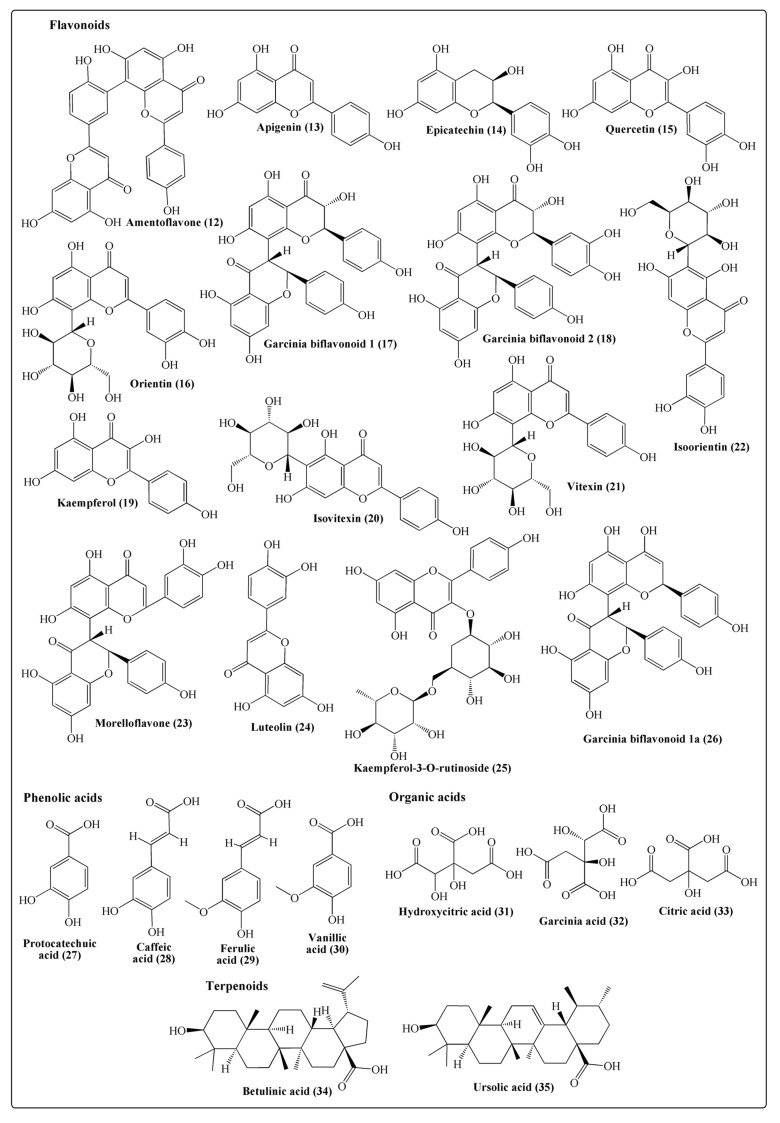
Structures of some flavonoids, phenolic acids, organic acids, and terpenoids isolated from *Garcinia morella.*

Flavonoids comprise a diverse group of polyphenolic compounds possessing a benzo-γ-pyrone structure that is abundant in all the plant species. Flavonols, flavones, flavanones, anthocyanins, isoflavones, and flavonols are the major classes of flavonoids that have been confirmed to possess a wide range of biological and therapeutic actions [20]. A total of 15 flavonoids (Figure 3) were isolated from the leaves of Indian gamboge, including amentoflavone (**12**), apigenin (**13**), epicatechin (**14**), Garcinia biflavonoid-1 (**17**), Garcinia biflavonoid-1a (**26**), Garcinia biflavonoid-2 (**18**), isoorientin (**22**), isovitexin (**20**), kaempferol (**19**), kaempferol-3-O-rutinoside (**25**), luteolin (**24**), orientin (**16**), quercetin (**15**), and vitexin (**21)** [3]. Morelloflavone (**23**) (Figure 3) was isolated and identified from heartwood [14]. *G. morella* possesses garcinia-biflavonoid-1 in optimal concentrations (399 ± 0.51 mg/g, Table 1), hence *G. morella* is a good source of this compound.

Phenolic acids are a class of natural compounds that are widespread throughout the plant kingdom and generally involved in a plethora of biological activities, namely antioxidant, anticancer, antidiabetic, antimicrobial, and hepatoprotective actions [21]. Phenolic acids are generally classified into benzoic acids containing seven carbon atoms (C6-C1) and cinnamic acids containing nine carbon atoms (C6-C3). Protocatechuic acid (**27**), caffeic acid (**28**), ferulic acid (**29**), and vanillic acid (**30**) (Table 1; Figure 3) were reported from the leaves of Indian gamboge. Protocatechuic acid concentration is highest in the leaves of *G. morella* (10.7 ± 0.57 mg/g) when compared to other major *Garcinia* species such as *G. indica* (0.407 ± 0.07 mg/g), *G. gummigutta* (0.427 ± 0.05 mg/g), and *G. mangostana* (1.2 ± 0.97 mg/g) [10]. *G. morella* is an excellent source of protocatechuic acid when compared to other well-known protocatechuic acid-yielding plants, namely *Olea europaea* (leaves, 3.23 ± 0.26 mg/g [22]), *Euterpe oleracea* (fruit, 0.63 ± 0.03 mg/g [23]), and *Boswellia dalzielii* (bark, 0.48 ± 0.00 mg/g [24]), and comparable to *Hibiscus sabdariffa* (calyx, 11.9 ± 1.2 mg/g [25]). Therefore, this plant is useful in the isolation of protocatechuic acid.

### 2.4. Organic Acids

Organic acids are synthesized in plants as a result of the incomplete oxidation of photosynthetic products and represent the stored pools of carbon accumulated due to different transient times of conversion of compounds in metabolic pathways [26]. Hydroxycitric acid (**31**), garcinia acid (**32**), and citric acid (**33**) (Figure 3; Table 1) were reported to be the major organic acids in the leaves of Indian gamboge [3,4]. Hydroxycitric acid is a derivative of citric acid found in the highest amount in *G. cambogia* fruits [27], and the majority of scientific evidence suggests that it possesses therapeutic value and promotes weight loss, suppresses de novo fatty acid synthesis, and increases lipid oxidation [28]. However, different case studies have demonstrated acute liver toxicity/failure in women consuming *G. cambogia* extract for weight loss. However, further studies are required on the use of herbal supplements involving hydroxycitric acid. Citric acid is considered as a valuable organic acid and widely used in the food, pharmaceutical, and cosmetic industries [29]. It is well-accepted as a safe food additive as evaluated by the FAO/WHO expert committee.

### 2.5. Terpenoids

Terpenoids are organic compounds derived from five-carbon isoprene units assembled and modified in different ways. Terpenoids are classified according to the number of isoprene units as monoterpenoids (C10), sesquiterpenoids (C15), diterpenoids (C20), sesterterpenoids (C25), and triterpenoids (C30) [30]. Monoterpenes consist of two isoprene units that may be linear or cyclic. They are abundantly available in essential oils and flavors. Ursolic acid (**34**) and betulinic acid (**35**) (Figure 3) are the two triterpenoids isolated from Indian gamboge (Table 1; [10]). Similarly, 16 volatile compounds, including allo-aromadendrene, aromadendrene, ascaridole, caryophyllene oxide, germacrene B, globulol, myrcene, selina-3,7(11) diene, spathulenol, α-copaene, α-humulene, β-caryophyllene, β-copaene, β-gurjunene, δ-amorphene, and δ-elemene, have been isolated from the leaves of Indian gamboge and are known to be present in leaves in different concentrations (Table 2, [31]).

### 2.6. Fatty Acids

Indian gamboge is rich in seed oil (38.08 g/100 g [32]), and it has been reported that the amount of oil present in the seeds of this plant is higher than the seed oil content of mangosteen [33]. The seed oil of Indian gamboge is used as a cooking oil and in confectionery [1]. The fatty acid composition of Indian gamboge was analyzed in seed oil [32,34] and stearic acid (44.95%) and oleic acid (45.38%) were identified as the major fatty acids; in addition, the oil contained myristic acid, palmitic acid, behenic acid, and heptadecanoic acid in smaller quantities (Table 3).

## 3. Biological Activities of Extracts and Compounds Isolated from *G. morella*

### 3.1. Antioxidant Properties

Table 4 summarizes the antioxidant properties of Indian gamboge fruit extracts and garcinol that have been examined. The antioxidant activities of water, acetone, and methanol extracts and that of a chloroform fraction containing garcinol have been demonstrated using 2, 2-diphenyl-1-picrylhydrazyl (DPPH) radical scavenging activity [35,36,37,38], hydrogen peroxide (H_2_O_2_) scavenging activity [38], the ferric thiocyanate method [35,36,38], and the 2, 2′-azino-bis-(3-ethylbenzthiazoline-6-sulfonic acid (ABTS) assay [37]. The total antioxidant activity (TAC) was demonstrated using the phosphomolybdate assay [36], nitric oxide radical inhibition assay [37], and cyclic voltammetry method [39]. The phytochemical analysis was conducted in water extracts of Indian gamboge fruits [38] and significant amounts of total phenolics (5.46 mg catechin equivalents/g) and total flavonoids (3.69 mg quercetin equivalents/g) were reported, which revealed a 2-diphenyl-1-picrylhydrazyl (DPPH) free radical scavenging activity (IC_50_) of 1.0 µg/mL and a H_2_O_2_-radical scavenging activity (IC_50_) of 1.33 µg/mL. Murthy et al. [40] investigated the phytochemicals of Indian gamboge resin/latex and reported that it consisted of 204.27 mg/g of phenolics and 124.92 mg/g of flavonoids. The resin exhibited significant antioxidant activities, with EC_50_ values of 205.5 µg/mL with DPPH, 95.53 µg/mL with phosphomolybdate, and 308.1 µg/mL with hydrogen peroxide scavenging assays.

These results demonstrate that the phenolics and flavonoids present in the fruit and resins are responsible for the antioxidant properties, along with other bioactive compounds. Indian gamboge is also rich in biflavonoids, xanthones, and benzophenones (Table 1) that act as antioxidants. Mangostins (α-, β-, and γ-mangostins) are xanthones reported in mangosteen (*G. mangostana*), and numerous in vitro and in vivo studies have reported that mangostins exhibit a wide range of pharmacological activities, including antioxidant properties [46]. William et al. [47] reported that α-mangostin acts as a free radical scavenger, reducing the oxidation of low-density lipoprotein (LDL) induced by copper or peroxyl radicals and decreasing the consumption of α-tocopherol induced by oxidized low-density lipoprotein (ox-LDL). α-Mangostin was isolated from the leaves of Indian gamboge [10] and it was reported that the antioxidant activities exhibited by the leaf extract might be due to mangostin, along with other bioactive compounds [35]. Garcinol was reported to be the principal compound, which is a tri-isoprenylatedchalcone, and exhibited efficient scavenging activity of DPPH, hydroxyl, methyl radicals, and superoxide anions [48]. Garcinol was also detected from *G. morella* by the authors of [36], who reported that the antioxidant activity exhibited by *G. morella* fruits and leaves is also due to garcinol.

### 3.2. Hepatoprotective Properties

There is evidence about the hepatoprotective properties of the fruit rind extract of *G. morella* against carbon tetrachloride (CCl_4_)-induced albino rats [37]. The hepatoprotective activity was determined by measuring the activity of liver function enzymes such as aspartate transaminase (AST), alanine transaminase (ALT), and alkaline phosphatase (ALP), and bilirubin and total protein in albino rats. It has been reported that albino rats treated with CCl_4_ showed elevated levels of liver function enzymes and bilirubin, but a suppressed production of total protein [37]. Pretreatment with the fruit extract significantly decreased the AST, ALT, ALP, and bilirubin levels and increased the production level of protein in a dose-dependent manner. The authors considered that the fruit rind extract of *G. morella* contains both phenolic and flavonoids that are responsible for the hepatoprotective activity. Wang et al. [49] demonstrated that γ-mangostin exhibited the most potent activity to attenuate tert-butyl hydroperoxide (*t*-BHP)-induced hepatocyte injury. At concentrations of 1.25 and 2.5 µg/mL, γ-mangostin was able to completely reverse the *t*-BHP-induced decreases in glutamine oxaloacetate transaminase and glutamate pyruvate transaminase levels in HL-7702 cells. Similarly, the authors of [50] reported that α-mangostin also has hepatoprotective activity and could significantly decrease the level of lipid peroxidation and decrease the levels of superoxide dismutase in a mouse model.

### 3.3. Anticancer Properties

Several studies have explored the anticancer activities of *G. morella* fruit, leaf, and bark extracts and different isolated compounds such as xanthones and benzophenones (Table 4). Choudhury et al. [41] investigated the anticancer activity of methanolic extracts of the leaf, bark, and fruit of *G. morella* under different in vitro and in vivo experimental conditions. Their study demonstrated that the fruit extract exhibited the maximum activity. The anticancer activity was further confirmed by the results of in vivo administration of the fruit extract (200 mg/kg) for 10 days to Dalton’s lymphoma-induced mice. The fruit extract significantly increased the mean survival time of the animals, decreased the tumor volume, and restored the hematological and biochemical parameters. The authors further showed that the fruit extract exerted its anticancer effect through the induction of caspases and DNA fragmentation that ultimately led to apoptosis. In another study, Choudhury et al. [36] confirmed the effect of *G. morella* methanolic fruit extract on breast cancer cell lines (MCF7, MDAMB231, and SKBR3). Their results of time-course analysis (at 24, 48, and 72 h) of bioactive fraction (1.56–25 µg/mL) treatment on breast cancer cell lines revealed a dose- and time-dependent antiproliferative response. Furthermore, mechanistic studies involving morphological observations and Western blotting analysis disclosed its apoptosis-inducing effect on breast cancer. P53-dependent upregulation of Bax and downregulation of B-cell lymphoma-extra large (Bcl X_L_) was suggested as the possible pathway of apoptosis followed by MCF7 cells upon exposure to the bioactive faction. Subsequently, through UHPLC and ESI-MS/MS analysis, Choudhury et al. [36] demonstrated that garcinol was the bioactive compound responsible for the anticancer activity. Hong et al. [51] investigated the effect of garcinol on the growth of HT-29 and HCT-116 colon cancer cells, as well as IEC-6 and INT-407 cells, which are normal immortalized intestinal cells. They demonstrated that garcinol exhibited potent growth inhibitory effects on all intestinal cells, with the IC_50_ values in the range of 3.2–21.4 µM after 72 h of treatment. Garcinol was found to be more effective in inhibiting the growth of cancer cells than inhibiting the growth of normal immortalized cells. In another study, Pan et al. [52] elucidated that garcinol suppressed the growth of human leukemia HL-60 cells by the induction of caspase-3/CPP32 activity and induction of the degradation of poly(ADP-ribose) polymerase (PARP) protein. More recent studies have demonstrated the activities of garcinol and isogarcinol against lung cancer, colorectal cancer, breast cancer, prostate cancer, pancreatic cancer, and cervical cancer models, and the activities were largely attributed to the inhibition of histone acetyl transferase (HATs), NF-*k*B signaling, and STAT signaling [53]. Several studies indicate that xanthones are cytotoxic against different types of cancer. For instance, desoxymorellin was found to inhibit the growth of human embryonic lung fibroblast and Henrietta Lacks cervical cancer cells, with a minimum inhibitory concentration of 0.39 µg/mL [54].

### 3.4. Anti-Inflammatory Properties

Inflammation is a biological response to the immune system that can be triggered by various factors, including pathogens, damaged cells, and toxic compounds. These factors may induce acute inflammatory responses in various organs, potentially leading to tissue damage or diseases. Both infectious and noninfectious agents activate inflammatory cells and trigger inflammatory pathways such as NF-kB, MAPK, and JAK–STAT pathways [55]. It has been reported that *G. morella* fruit is a good source of antioxidant and anti-inflammatory agents [36], and the anti-inflammatory assay showed that it significantly decreased the release of nitrate and TNF-α levels of lipopolysaccharide-induced RAW 246.7 cells. It has been reported that treatment of carrageenan-induced paw edema rats with 20 mg/kg of *G. morella* methanolic fruit extract containing garcinol significantly inhibited paw inflammation and controlled the cytokine and nitrate levels of the induced-edema rats [36]. Several researchers have investigated and confirmed the anti-inflammatory activities of mangostin isolated from several *Garcinia* species. Mangostin attenuated the lipopolysaccharide (LPS)-induced expression of inflammatory mediators such as tumor necrosis factor α (TNF-α) and interleukin 6 (IL-6) in human U939 macrophage-like cells. Mangostin also decreased the activation of several signaling pathways, including IL-1, mitogen-activated protein kinase (MEK), JNK, ERK, signal transducer and activation of transcription 1 (STAT-1), and activator protein 1 (AP-1), in these cells [56,57]. All the above-described data indicate that garcinol and mangostin could be novel targets for anti-inflammatory compounds.

### 3.5. Antimicrobial Properties

Several studies have demonstrated the antimicrobial (antibacterial, antifungal, and antiviral) properties of extracts and isolated compounds obtained from Indian gamboge (Table 4). Narasimha Rao et al. [58] examined the antibacterial and antifungal properties of morellins and found that *Micrococcus pyogenes* var. *aureus*, *Mycobacterium phlei*, and *M. tuberculosis hominis* were susceptible to the methyl ether of morellin. Similarly, morellin, morellin-T, morellin-M, morellin-L, and isomorellin, which were isolated from *G. morella*, demonstrated antibacterial activity against Gram-positive and -negative bacteria, fungi, yeast, and actinomycetes [59]. The antibacterial activity of leaf methanolic extracts of nine *Garcinia* species [35], including *G. morella*, against *Escherichia coli* (MTCC 441), *Bacillus cereus* (MTCC 430), *Staphylococcus aureus* (MTCC7433), *Salmonella enterica* ser. *typhi* (MTCC733), and *Vibrio cholera* (MTCC 3906), was evaluated and it was found that *G. morella* methanolic extract exhibited remarkable antibacterial activity against *B. cereus* (MTCC 430) and *S. aureus* (MTCC7433) at 200 and 500 µg/mL concentrations. Similarly, Sarma et al. [38] demonstrated the antifungal activities of fruit extracts of *G. morella* against skin pathogenic fungi, namely *Trichophyton rubrum*, *Microsporum gypseum*, and *Microsporum fulvum* (Table 4). Zuo et al. [45] investigated the in vitro antimicrobial activities of 80% ethanol extracts of 30 Chinese medicinal plants, including *G. morella*, against conventional clinical pathogens such as *S. aureus* (ATCC 25923, methicillin-sensitive *Staphylococcus aureus* (MSSA)), *E. coli* (ATCC 25922), *Pseudomonas aeruginosa* (ATCC 27853), and *Candida albicans* (ATCC Y0109) by agar diffusion method and calculated the inhibition zone diameters (IZDs). The screening of the in vitro antimicrobial activity of *G. morella* extracts revealed the anti-MSSA (IZD 17 mm) and anti-MRSA (methicillin-resistant *S. aureus*) (IZD 15.7 mm) effects at various levels of potency, and the authors reported that the anti-MRSA activity of *G. morella* was due to morellin and other caged xanthones. Numerous in vitro and in vivo studies have demonstrated that mangostin (**10**) exhibited antibacterial, antifungal, and antimalarial properties [46]. Another study reported that α-mangostin exhibited inhibitory effects against MRSA and vancomycin-resistant *Enterococci* (VRE [60]), wherein the authors showed that α-mangostin is involved in the disruption of the bacterial cytoplasmic membrane at minimum inhibitory concentrations of 0.78–1.56 µg/mL. Similarly, previous studies [61,62,63,64] reported the inhibitory effects of α-mangostin against *Streptococcus mutans*, *Enterococcus faecalis*, *Mycobacterium tuberculosis*, *Plasmodium falciparum*, and *Plasmodium berghei*. Garcinol (**11**) also demonstrated significant activity against disease-causing microbes, namely influenza A [65], *Bacillus anthracis* [66], *C. albicans* [67], and *Toxoplasma gondii* [68]. Garcinol was reported to inhibit both the viral nucleoprotein and regulation of the viral polymerase of influenza A.

### 3.6. Larvicidal Properties

Insect vectors, especially mosquitoes, directly transmit human diseases such as filarial fever, malaria, dengue fever, and chikungunya, among others [69]. One of the strategies to control these vectors is to destroy their vectors and intermediate hosts. The use of natural products for the control of insect pests offers an economically viable and ecofriendly approach. In recent years, plants have been identified for their insecticidal or larvicidal properties and used to control insect vectors. Twenty-five plant extracts were screened [44], including *G. morella*, for larvicidal activity against *Culex quinquefasciatus* Say. (third instar larvae) at 100-ppm concentration and the larval mortality was evaluated after 24 and 48 h. Their experimental results revealed that hexane and dichloromethane extracts of *G. morella* extract were responsible for 100% of the mortality of *C. quinquefasciatus* larvae. Murthy et al. [40] investigated the phytochemical composition of the resin/latex of *G. morella* and evaluated the larvicidal activity (latex was dissolved in 1 mL of acetone, and different concentrations, namely 37.5, 75, 150, 300, and 600 ppm, were prepared) against the filariasis-causing vector *C. quinquefasciatus*. They reported that *G. morella* resin/latex exhibited toxicity against the treated third instar larvae of *C. quinquefasciatus*, with LC_50_ and LC_90_ values of 132.54 and 483.15 ppm, respectively. The bioactive compounds isomorellin (**7**), morellic acid (**4**), and isomorellic acid (**6**) of *G. morella* resin [8,12] might be responsible for the larvicidal activities.

## 4. Conclusions

This review presents a comprehensive account of the phytochemical constituents and biological activities of *G. morella*. The fruits of this plant are edible, and the oil/fat obtained from its seeds is used as edible oil or ghee. Different secondary metabolites, such as xanthones, benzophenones, flavonoids, phenolic acids, organic acids, and terpenoids, have been isolated from the fruits, leaves, seeds, and heartwood of *G. morella*, which have demonstrated several biological activities, including antioxidant, hepatoprotective, anticancer, anti-inflammatory, antimicrobial, and larvicidal properties. These properties suggest that *G. morella* is an important source of therapeutic compounds. Nevertheless, additional research efforts are required to evaluate the toxicity of the phytochemicals isolated from this plant. There is also a need for research to explore the novel bioactive compounds of this valuable plant.

## Figures and Tables

**Table 1:** Chemical compounds isolated from *Garcinia morella.*

Plant Part/Exudates	Chemical Group	Chemical Compounds	Presence (mg/g) *	References
Leaf	**Xanthones**	Gambogic acid	1.79 ± 0.36	[3]
		Mangostin	0.085 ± 0.21	
	**Benzophenone**	Garcinol	0.318 ± 0.17	
	**Flavonoids**	Amentoflavone	2.51 ± 0.23	
		Apigenin	0.724 ± 1.03	
		Epicatechin	0.218 ± 0.30	
		Garcinia biflavonoid 1	399 ± 0.51	
		Garcinia biflavonoid 1a	22.1 ± 0.15	
		Garcinia biflavonoid 2	6.14 ± 1.01	
		Isoorientin	1.32 ± 1.05	
		Isovitexin	3.55 ± 1.02	
		Kaempferol	0.289 ± 0.07	
		Kaempferol-3-O-rutinoside	0.006 ± 0.02	
		Luteolin	0.588 ± 0.07	
		Orientin	2.21 ± 0.07	
		Quercetin	0.238 ± 0.03	
		Vitexin	2.16 ± 0.75	
	**Phenolic acids**	Caffeic acid	0.595 ± 0.06	
		Ferulic acid	0.191 ± 0.03	
		Protocatechuic acid	10.7 ± 0.57	
		Vanillic acid	0.001 ± 0.05	
	**Organic acids**	Hydroxycitric acid	3.55 ± 0.55	
		Garcinia acid	6.46 ± 1.20	
		Citric acid	2.90 ± 0.00	[4]
	**Triterpenoids**	Betulinic acid	1.83 ± 0.11	[3]
		Ursolic acid	1.25 ± 0.07	
Seed	**Xanthones**	Desoxymorellin	NR **	[5]
		Dihydroisomorellin	NR	[5]
		Morellin	NR	[6,7]
		Morellinol	NR	[8]
		Moreollin	2.40 ± 0.00	[9]
Resin	**Xanthones**	Gambogic acid	NR	[10,11]
		Isomorellic acid	NR	[11]
		Isomorellin	NR	[12]
		Morellic acid	NR	[11]
Fruit	**Benzophenone**	Garcinol	0.0072 ± 0.00	[13]
Heartwood	**Flavonoids**	Morelloflavone	NR	[14]

* mean ± SD, *n* = 3; ** NR = not reported.

**Table 2:** Volatile compounds isolated from the leaves of *Garcinia morella* [31].

Volatile Compounds	Amount (%)
Alloaromadendrene	0.1
Aromadendrene	2.8
Ascaridiole	0.1
Caryophyllene oxide	6.7
Garmacrene B	0.8
Globulol	1.9
Myrcene	0.1
Selina-3,7(11) diene	0.2
Spathulenol	0.1
α-Copaene	1.3
α-Humulene	18.5
β-Caryophyllene	0.1
β-Copaene	49.4
β-Gurjunene	0.1
δ-Amorphene	0.5
δ-Elemene	0.1

**Table 3:** Fatty acid composition of the seed oil of *Garcinia morella.*

Fatty Acids	Composition (%)	
	[32]	[34]
Myristic acid	0.02	ND *
Palmitic acid	1.04	0.7
Palmitoleic acid	0.02	ND
Margaric acid	0.12	ND
cis-10-Heptadecanoic acid	0.02	ND
Stearic acid	44.95	46.4
Oleic acid	45.38	49.5
Linoleic acid	7.77	0.9
Linolenic acid	0.09	ND
Arachidic acid	0.31	2.5
Behenic acid	0.24	ND
Total saturated fatty acids	46.70	49.6
Total unsaturated fatty acids	53.29	50.4

* ND = not detected

**Table 4:** Biological activities of extracts and compounds isolated from *Garcinia morella.*

Part/Resin	Extract/Compound	Activity	Cell Lines/Models	References
Fruit	Methanol and chloroform fraction containing garcinol	Antioxidant and anticancer activities	Breast cancer cell lines (MCF7, MDAMB231, and SKBR3)	[36]
	Methanol	Antioxidant and hepatoprotective activities	CCl_4_-induced hepatic injury	[37]
	Water	Antioxidant and antifungal activities	*Trichophytonrubrum*, *Microsporumgypseum*, and *Microsporumfulvum*	[38]
	Methanol	Antioxidant activity	The change of oxidation potential in the redox cycle of 1,4-diaminobenzene	[39]
	Garcinol	Anticancer activity	Neuroblastoma cell line (SH-SY5Y)	[13]
Fruit, bark and leaf	Methanol	Anticancer activity	T-cell murine lymphoma	[41]
Latex/resin	Acetone	Antioxidant and larvicidal activities	*Culexquinquefasciatus*	[40]
Leaf	Ethanol	Anticancer activity	Human colon, oral cancer, human ovary, breast cancer, and liver cancer cell lines	[42]
	Methanol	Axiolytic activity	Wistar albino mice	[43]
	Hexane	Larvicidal activity	*Culex quinquefasciatus*	[44]
Whole plant	Ethanol	Antimicrobial	*Staphylococcus aureus*, *Escherichia coli*, *Pseudomonas aeruginosa*, and *Candida albicans*	[45]

## Abbreviations

ABTS, 2, 2′-azino-bis-(3-ethylbenzthiazoline-6-sulfonic acid); ALP, alkaline phosphatase; AST, aspartate transaminase; ALT, alanine transaminase; ATCC, American Type Culture Collection; caspase-3, cysteine-aspartic acid protease; CBP32, cysteine-aspartic acid protease 32; CCl_4_, carbon tetrachloride; DPPH- 2, 2-diphenyl-1-picrylhydrazyl; EC_50_, half maximal effective concentration; ESI-MS/M, electron spray ionization mass spectroscopy; FAO, Food and Agriculture Organization of the United Nations; HCT-116, human colon colorectal carcinoma cell line; HL, normal human liver cells; H_2_O_2_, hydrogen peroxide; HL, normal human liver cells; HT-29, human colorectal adenocarcinoma cell line; HAT, histone acetyl transferase; IC_50_, concentration of a drug/chemical that is required for 50% inhibition in vitro; IEC-6, rat small intestine epithelium cell line; IL-1, interleukin group of cytokines; INT-407, human intestinal epithelial cell line; IZD, inhibition zone diameter; JAK–STAT, Janus kinases-signal transducer and activator of transcription proteins; LC_50_, lethal dose at which 50% of the population is killed in a given period of time; LDL, low-density lipoprotein; LPS, lipopolysaccharide; MCF7, human breast cancer cell line; MDA-MB231, human breast adenocarcinoma cell line; MSSA, methicillin-sensitive *Staphylococcus aureus*; MRSA, methicillin-resistant *Staphylococcus aureus*; MTCC, Microbial Type Culture Collection; NF-*k*B, nuclear factor kappa-light-chain-enhancer of activated B cells; PART, poly(ADP-ribose) polymerase protein; ppm, parts per million; RAW, functional macrophage cell lines transformed by Abelson leukemia virus; SKBR3, human breast cancer cell line; TAC, total antioxidant activity; *t*-BHP, tert-butyl hydroperoxide; TNF-α, tumor necrosis factor alpha; UHPLC, ultra-high-performance liquid chromatography; VRE, vancomycin-resistant *Enterococci*; WHO, World Health Organization.
