# Erratum: Murthy et al. Phytochemicals and Biological Activities of *Garcinia morella* (Gaertn.) Desr.: A Review. *Molecules* 2020, *25*, 5690

**DOI:** 10.3390/molecules26154398

**Published:** 2021-07-21

**Authors:** Hosakatte Niranjana Murthy, Dayanand Dalawai, Yaser Hassan Dewir, Abdullah Ibrahim

**Affiliations:** 1 Department of Botany, Karnatak University, Dharwad 580003, India; hnmurthy60@gmail.com (H.N.M.); dayananddalawai@gmail.com (D.D.); 2 Plant Production Department, College of Food and Agriculture Sciences, King Saud University, P.O. Box 2460, Riyadh 11451, Saudi Arabia; adrahim@ksu.edu.sa; 3 Faculty of Agriculture, Kafrelsheikh University, Kafr El-Sheikh 33516, Egypt

The authors wish to make the following change to the funding section in their paper [1]. The original funding section in the published paper “The authors extend their appreciation to the Deputyship for Research & Innovation, Ministry of Education, Saudi Arabia, for funding this research work through the project number IFKSURP-59. H.N.M. is thankful for the University Grants Commission-Basic Scientific Research (UGC-BSR) mid-career award grant (No. F.19-223/2018/ (BSR)” is corrected as follows:

The authors extend their appreciation to the Deputyship for Research & Innovation, Ministry of Education, Saudi Arabia, for funding this research work through the project number IFKSURP-59.

We apologize for any inconvenience caused to the readers by this mistake.

## References

[1] Murthy H.N., Dalawai D., Dewir Y.H., Ibrahim A. (2020). Phytochemicals and Biological Activities of *Garcinia morella* (Gaertn.) Desr.: A Review. Molecules.

